# Supplementary material for: Managing well-being in paediatric critical care: a multiperspective qualitative study of nurses’ and allied health professionals’ experiences
Source: BMJ Open. 2024 May 28;14(5):e084926. doi: 10.1136/bmjopen-2024-084926 (PMC11138289; doi:10.1136/bmjopen-2024-084926)
Supplement: Author's manuscript [file bmjopen-2024-084926.draft_revisions.pdf]

The pre-publication history for this article, or part of it, is not displayed.

BMJ Open aims to promote transparency in the publication process by publishing reviewer reports and previous versions of manuscripts as pre-publication histories. This means that accepted papers usually have their prepublication history posted alongside them. This comprises all previous versions of the manuscript, the reviewers' comments, and the authors' responses to all the comments from reviewers and editors. For clinical trials, this also includes the study protocol.

However, in a small number of cases, after careful consideration, we decide not to publish the pre-publication history, or part of it.

That decision has been taken in respect of this article.

We do not provide reasons in specific cases. However, examples could include cases involving stigmatized illnesses raising issues of reviewer confidentiality and data protection, or where there are legal or regulatory considerations. Whatever the reason, the decision has no bearing at all on the quality or integrity of the prepublication process.

In all cases where we have determined that the prepublication record, or part of it, should not be publicly available, we expect that authors will respect this decision and likewise refrain from sharing it.
